# Impact of the COVID-19 Pandemic on Physical Activity and Sedentary Behaviour: A Qualitative Study in a Canadian City

**DOI:** 10.3390/ijerph18094441

**Published:** 2021-04-22

**Authors:** Jennie A. Petersen, Calli Naish, Dalia Ghoneim, Jason L. Cabaj, Patricia K. Doyle-Baker, Gavin R. McCormack

**Affiliations:** 1 Department of Community Health Sciences, Cumming School of Medicine, University of Calgary, 3280 Hospital Drive NW, Calgary, AB T2N 4Z6, Canada; carol.naish1@ucalgary.ca (C.N.); dghoneim@ucalgary.ca (D.G.); Jason.Cabaj@albertahealthservices.ca (J.L.C.); Gavin.McCormack@ucalgary.ca (G.R.M.); 2 Faculty of Applied Health Sciences, Brock University, 1812 Sir Isaac Brock Way, St. Catharines, ON L2S 3A1, Canada; 3 Department of Communication, Media and Film, Faculty of Arts, University of Calgary, 2500 University Drive NW, Calgary, AB T2N 1N4, Canada; 4 Provincial Population & Public Health, Alberta Health Services, 10301 Southport Lane SW, Calgary, AB T2W 1S7, Canada; 5 Faculty of Kinesiology, University of Calgary, 2500 University Drive NW, Calgary, AB T2N 1N4, Canada; pdoyleba@ucalgary.ca; 6 School of Planning, Architecture, and Landscape, University of Calgary, 2500 University Drive NW, Calgary, AB T2N 1N4, Canada; 7 Faculty of Sport Sciences, Waseda University, 1-104 Totsukamachi, Shinjuku-ku, Tokyo 169-8050, Japan

**Keywords:** pandemic, COVID-19, coronavirus, physical activity, sedentary behaviour, health, qualitative

## Abstract

Public health measures introduced to combat the COVID-19 pandemic have impacted the physical activity, health, and well-being of millions of people. This grounded theory study explored how the COVID-19 pandemic has affected physical activity and perceptions of health among adults in a Canadian city (Calgary). Twelve adults (50% females; 20–70 years) were interviewed between June and October (2020) via telephone or videoconferencing. Using a maximum variation strategy, participants with a range of sociodemographic characteristics, physical activity levels, and perceptions of seriousness and anxiety related to COVID-19 were selected. Semi-structured interviews captured participant perceptions of how their physical activity and perceptions of health changed during the pandemic. Using thematic analysis, four themes were identified: (1) Disruption to Daily Routines, (2) Changes in Physical Activity, (3) Balancing Health, and (4) Family Life. Participants experienced different degrees of disruption in their daily routines and physical activity based on their individual circumstances (e.g., pre-pandemic physical activity, family life, and access to resources). Although participants faced challenges in modifying their daily routines and physical activity, many adapted. Some participants reported enhanced feelings of well-being. Public health strategies that encourage physical activity and promote health should be supported as they are needed during pandemics, such as COVID-19.

## 1. Introduction

On 11 March 2020, the World Health Organization (WHO) declared coronavirus disease (COVID-19) a pandemic [1]. As of 12 March 2021, there have been over 118 million total confirmed COVID-19 cases and more than 2,621,000 related deaths globally. Across Canada, 896,739 COVID-19 cases have been confirmed, with 22,335 related deaths, and counts will likely continue to increase [2]. In response to the pandemic, governments worldwide, including Canada, have implemented public health emergency measures to reduce the spread of COVID-19. These restrictions involve legally enforced closures, and/or limitations on the maximum capacity of destinations where people congregate (e.g., schools, recreational facilities, and shops) [3]. Additional mandatory and voluntary measures that have disrupted pre-pandemic routines consist of physical distancing, mask wearing, restrictions on non-essential international and domestic travel, and self-isolation/quarantine requirements [3,4]. The Alberta Government (Canada) declared a state of public health emergency on 15 March 2020 and implemented a 3-month closure of sport programs, fitness centers and recreation facilities [5]. These restrictive measures may have negatively impacted physical activity levels [6].

### 1.1. Impact of COVID-19 on Physical Activity and Sedentary Behaviour

Public health responses to infectious disease pandemics, such as COVID-19, have the potential to impact the daily physical activity levels of millions of people [7,8]. Sedentary behaviour includes activities such as sitting, reclining, watching TV, playing video games and use of a computer [9], and is associated with negative health outcomes [10]. Prolonged reductions in physical activity and increases in sedentary behaviour, especially for extended periods of time, could lead to deleterious effects on cardio-metabolic [11,12,13] and mental health outcomes [14,15]. Decreases in physical activity and increases in sedentary behaviours can put individuals at increased risk of chronic health conditions (e.g., CVD, type 2 diabetes, obesity, hypertension, and depression) [16]. From the opposite perspective, moderate physical activity strengthens the immune system [17] and can protect against respiratory infection [18,19]. The World Health Organization [20] recommends that adults participate in at least 150–300 min of moderate or 75–150 min of vigorous physical activity per week and reduce their time spent in sedentary behaviour to achieve health benefits.

Recent quantitative studies undertaken in Canada [21], Europe [22], China [23], and the United States [24] identified changes in physical activity and sedentary behaviour due to COVID-19. A cross-sectional study conducted in Canada found that when compared to pre-COVID-19 physical activity levels, adults perceived a decrease in their weekly moderate-to-vigorous-intensity activity despite 58% still accumulating the recommended levels of physical activity [21]. In France and Switzerland, enforced lockdowns have been associated with increases in walking, moderate-intensity physical activity and leisure-based sedentary behaviour [22]. In the U.S., adults have reported decreases in physical activity and increases in sedentary behaviour [24]. Despite consistent findings in regard to increased sedentary behaviour, impacts of the pandemic on physical activity are equivocal.

### 1.2. Adaptation and Behaviour Change

Shifts towards alternative forms of physical activity during the COVID-19 pandemic might be necessary for adults to maintain benefits of their regular routines [6,25]. Life events such as marriage, pregnancy, and retirement are known to impact physical activity routines. However, these transitions are often planned [26]. Few studies have explored how people have adapted their behaviour, including the facilitators and/or barriers to physical activity and sedentary behaviour, during the COVID-19 pandemic [6,27]. Several theories exist to predict changes in health behaviour [28], although these theories were not created within the context of pandemic conditions. Individual behaviour change theories such as the Health Belief Model [29], Transtheoretical Model of Change [30], and Theory of Planned Behaviour [31] suggest that beliefs, attitudes, expectations, self-efficacy, and intentions are important factors within health behaviour change processes [32]. In contrast, socioecological models posit that there are reciprocating factors at multiple levels (e.g., cultural, policy, legislative, environmental, interpersonal, and intra-personal) that influence health behaviours, such as physical activity [33,34]. An individual’s environment should be considered in the development of effective intervention strategies [35], especially during crises [36]. The pandemic has created a unique opportunity to explore how people adapt their physical activity and health behaviours during unforeseen circumstances in which individuals are facing challenging restrictions to their movement.

The purpose of this qualitative study was to explore how the COVID-19 pandemic has affected physical activity, sedentary behaviour and perceptions of health among adults. The study objectives were to: (1) describe how physical activity and sedentary behaviour have been impacted by the public health response to COVID-19 in Calgary (AB, Canada); and (2) explore perceptions of health in relation to changes in physical activity during the pandemic.

## 2. Materials and Methods

### 2.1. Study Design

This qualitative study was undertaken in Calgary (AB, Canada) and involved semi-structured interviews. A grounded theory approach guided the study design, with the aim of describing the lived experiences of adults during the pandemic [37]. Grounded theory studies [37] often focus on social processes or actions [38,39]. Constructivist grounded theory approaches place emphasis on the shared meaning constructed by both the participant(s) and the researcher [38]. Within this approach, questions often center on what happens and how people interact in relation to the phenomenon [38,39]. The constructivist grounded theory approach suited our study as we sought to understand what happened to people and how they responded during the pandemic [38,39]. As is common with grounded theory approaches, sampling, data collection and data analysis are integrally related and iterative [38]. The University of Calgary Conjoint Human Research Ethics Board approved this study (REB 20-0854).

### 2.2. Sampling and Recruitment

Twelve participants (6 males and 6 females, aged 20–70 years) who previously participated in a community survey [40] were recruited for this qualitative study. Briefly, between April and June 2020, a random sample of adults (≥18 years of age) residing in north central Calgary were invited to complete an online questionnaire. The questionnaire was conducted as part of a community-based evaluation study taking place in north central Calgary and captured information about perceptions and participation in outdoor play and physical activity among families, social connections, and neighbourhood satisfaction, as well as responses to COVID-19 [40]. For our qualitative study, we recruited from the 1124 questionnaire participants who affirmed their willingness to participate in future research. An email was sent to eligible respondents inviting them to participate in an interview.

Given the far-reaching effects of the COVID-19 pandemic, we theorized that people’s physical activity experiences and their perceptions of health during the pandemic would differ based on multiple factors. These included sociodemographic background, and perceived seriousness and levels of anxiety towards COVID-19. Aligned with a grounded theory approach, we sought heterogeneity in our sample to develop a robust theoretical understanding of what has happened to people’s physical activity during the COVID-19 pandemic and how it has affected their perceptions of health [36]. A maximum variation sampling strategy was used to ensure we captured a range of perceptions and experiences with regard to physical activity and health during the pandemic [41,42]. Our sample involved participants with varied sociodemographic backgrounds, and a range of annual household income levels (<$40,000 to ≥120,000) and education levels (high school completion to a graduate degree). We also selected participants with different levels of perceived seriousness (perceived COVID-19 as ‘not serious’ to ‘extremely/very serious’) and different levels of anxiety (‘not anxious’ to ‘extremely/very anxious’ about COVID-19) related to the COVID-19 pandemic.

### 2.3. Data Collection

In this study, similar to many qualitative traditions, the data collection and analysis were conducted simultaneously [43]. Individuals who were interested in this study were invited to participate in a semi-structured interview over telephone or video conference using Zoom. Prior to the interview, participants were emailed a document explaining this study and the informed consent process. Participants were asked for verbal consent and permission to audio record at the start of their interview. Interviews lasted approximately 50 min. Two interviewers (first and second author) trained in qualitative methodology conducted the interviews. Both interviewers recorded detailed memos, with notes throughout and after the interviews to support reflexivity [43]. Reflexivity was addressed by examining how the interviewers and participants monitored and responded to each other’s talk throughout. For example, when they received empathetic and interested responses from the interviewers, participants often responded by becoming more open, providing more depth about their experiences [44].

The Government of Alberta began to lift certain public health restrictions and re-open previously closed public spaces during the timeframe that interviews were undertaken. For example, most retail businesses (e.g., clothing stores, bookstores) and restaurants were able to resume operations as of 14 May 2020, while recreation and fitness centers were permitted to re-open on 12 June 2020 [45]. Physical distancing measures and restrictions to social gatherings (i.e., maximum gathering of 15 people indoors and 50 people outdoors) remained in place for the duration of this study [45].

A semi-structured interview guide, informed by the socioecological model, was used to collect the thoughts of participants while providing flexibility to capture information about a broad topic that had emergent directions depending on the participant experiences [43]. Participants were asked to describe how their physical activity and perceptions of health changed in response to the local COVID-19 public health measures, and to reflect on their attitudes towards physical distancing. Finally, participants were asked what they found most challenging about staying physically active and what they discovered about their health during the pandemic. Interviewed participants received a $50 gift card as a token of appreciation.

Braun and Clark [46] discuss the importance of the depth of engagement with the data and reflexive interpretation in considering data saturation, beyond the prevalence of certain codes or themes constructed from the data. This approach to data saturation aligns with a constructivist grounded theory methodology [46]. Our sample size of 12 participants was deemed sufficient given the depth and breadth of the data collected, in conjunction with reflexive interpretation of the data and our own lived experiences during the pandemic.

### 2.4. Data Analysis

Thematic analysis [47] was used to explore the patterns and commonalities that people were having in connection to their physical activity and perceived health during the pandemic. Our six steps for thematic analysis involved: (1) familiarizing ourselves with the data, (2) generating initial codes, (3) searching for themes, (4) reviewing themes, (5) defining and naming themes and (6) writing the manuscript [47]. Interview data were transcribed verbatim and analysis occurred after each interview. To support confirmability of the data, all participants were invited to review a de-identified version of their transcript [38,40]. Four participants reviewed their transcript post-interview. Participant names were replaced with pseudonyms to ensure anonymity.

The interviewers reviewed all transcripts, then organized and coded data using NVivo version 12 [48]. To ensure trustworthiness, the interviewers met regularly to discuss the meaning of the codes and themes generated in relation to the research questions [42]. Moreover, to strengthen dependability of results, codes and themes were discussed and interpreted among the research team (all co-authors) [38,40]. Recorded memos and notes were organized in NVivo and an audit trail of the data analysis process was kept to enhance trustworthiness [38,40].

### 2.5. Reflexivity

To support trustworthiness in our study, reflexivity, a fundamental practice used in qualitative research to critically reflect on how the researcher influences the research process was used. [43]. The participants described disruptions they were experiencing related to the COVID-19 public health measures—experiences shared by the research team. Our research team meetings where data interpretation began to take place were often intertwined with reflections about our own lived experiences during the pandemic. Experiencing a phenomenon while also studying it sensitized us to continuously examine our own views on the research topic [44]. For example, our research team was surprised by the positive experiences some participants were having during the pandemic and this challenged us to question our perspectives about the public health restrictions. Furthermore, moments where our interviewers shared aspects themselves during an interview supported reciprocity, helping to build rapport and trust with participants [44]. The act of reflecting on participant interviews, including our similarities and differences, shaped our own views, helped bring awareness to our own assumptions and how we as researchers shaped the research process.

## 3. Results

All codes were organized into broad categories before identifying four main themes associated with the COVID-19 pandemic: (1) Disruption to Daily Routines; (2) Changes in Physical Activity; (3) Balancing Health, and; (4) Family Life.

### 3.1. Theme #1: Disruption to Daily Routines

The pandemic disrupted the activities that participants engaged in and the time spent engaging in these activities during work, school, home and social life, leisure and play. Participants described extensive changes and adaptations to their daily routines. Notably, many participants reported having extra time in their day as an unanticipated benefit of the pandemic.

#### 3.1.1. Adaptations to Daily Routines

Participants described ways in which they adapted their daily routines to a ‘new normal’ that incorporated public health measures, including physical distancing. As Beverly, highlights,

*“I had to learn how to create my own structure and that was a little difficult. I tried to create normalcy by [going to the library], and then the library shut down… now I work from home, so a lot of my schedule is constructed by work. And honestly, I don’t know what I’d be doing if I wasn’t working. That’s given me the structure to at least wake up a bit early”.*

Some participants initially noted little change in their routine, but later described several changes they had made. For example, John initially described life as “pretty much status quo”, but later described how he had increased spending his time at home. Aside from participants who were retired, adaptations with work or school were necessary due to restrictions at the office or requirements to work from home. Christy explained how she adapted to her new work routine,

*“I start my workday now earlier than I used to. I still work really long days and the work-life balance is not very good right now. it’s hard to stop work at 5:00… it was getting really bad where I am working from 7:00 AM till midnight with very little break. Now I’ve tried to put more restrictions on my work but we’re getting to take the kids for more walks throughout the day and play outside when I have breaks between meetings”.*

A sense of boredom and idleness was also experienced by many participants, as Arun suggests,

*“… having nothing to do… sitting idly... watching television, on your mobile phone… life has stopped”.*

The extent to which COVID-19 disrupted the structures of daily schedules varied between participants. While significant setbacks and challenges were experienced, participants also encountered benefits from these routine changes.

#### 3.1.2. Unanticipated Benefits of COVID-19

Participants noted how their perceptions of time had changed due to the pandemic-related restrictions. For example, Christy, was required to work from home when the pandemic began. She described the extra time gained and how this positively affected her daily routine,

*“Before the pandemic started, my husband and I worked at the same office, but we had to commute. We have two kids in daycare, both under the age of five… We would rush to get the house ready and get everybody out the door every morning with super stress and scrambled to drop the kids off to daycare for 7:45. Then we would drive an hour to work. Then I would work like crazy and work through my lunch hours and would never have a moment to breathe… we’ve gained this time from not commuting”.*

Madison also described the initial changes she encountered as a post-secondary student living out of province and how she used her time during the pandemic,

*“I was in New Brunswick for school, so I had to come back [to Calgary]. So that was a bit of a shit show over there at school. But when I came back, honestly, I’ve kind of been enjoying it… I’ve been doing a lot of homework… I’m doing four classes right now. I’m getting lots done”.*

The shifts in daily routine due to the COVID-19 pandemic changed how participants spent their time and this was perceived by some as a positive benefit.

### 3.2. Theme #2: Changes in Physical Activity and Sedentary Behaviour

The province-wide public health emergency measures initiated in mid-March 2020, required fitness centers, recreational facilities, and sports clubs in Calgary to halt operations for approximately three months, after which they could operate with public health restrictions in place, creating interruptions to participants’ usual physical activity routines.

#### 3.2.1. Disruptions to Physical Activity

Participants spoke about the challenges of finding “equivalent” activities to maintain their physical activity routines (e.g., weight training, swimming, and fitness classes) without access to fitness, sport, and recreation facilities. Allan explained how his workout routine was negatively impacted,

*“Probably the biggest thing is I do go to the gym just about every day, so my gym closed… I was looking at alternatives to keep active, but I did miss my weights. I don’t own any heavy weights and usually I like the circuit machinery at the gym for a part of my workout. Not having access to that definitely altered my workouts considerably”.*

Mya, a regular aquacise participant prior to the pandemic, also experienced negative physical changes in her body and reductions in her strength,

*“I really notice my upper muscles above my knee are getting weaker”.*

Participants who were laid off or forced to work or study from home increased their sedentary behaviour due to less time spent walking during their workday (e.g., from their vehicle to the office; between meetings or classes). Madison elaborated on this in her interview,

*“… at school, you’re moving a lot throughout the day… here I sit inside for a couple hours, then I go for my two hour period of exercise… then I’m sitting inside again for the rest of the day”.*

Time spent in sedentary activities also increased since the pandemic for some participants like John who described watching more television to help pass the time,

*“… we watch a lot more Netflix than we ever have… We’ve probably doubled… you can watch one, two, three, four, and five, next thing you know it’s eight hours later”.*

While numerous challenges were discussed, other participants had become more physically active during the pandemic. Christy, who started working from home, explained,

*“I have an Apple Watch. It tracks your steps and active movement. It also registers when you’re going up a level and it’s considered true exercise. I seem to be getting more exercise credit than I was at the office. I’m obviously crushing myself slightly harder on those longer walks, or on the bike rides versus walking from meeting to meeting. I think that’s good”.*

#### 3.2.2. Adaptations to Physical Activity Routines

Participants appreciated the opportunity to try new physical activities. For example, Beverly explained how she bought a skateboard, something she had never done before. Some participants reported using online workout videos, purchasing at-home fitness equipment, and using pathways more often for walking or cycling. Other participants initiated modified versions of the physical activities that they had previously undertaken at now closed facilities. Ana described setting up a yoga studio in her home,

*“I don’t think I’ll go back to yoga class anymore because I discovered a new way to do yoga without spending money for gym and yoga class”.*

Similarly, Jason moved to doing cardio exercise outdoors and describes the benefits he experienced,

*“I took up running around the block... the first couple of times I was huffing and puffing like an old man, but… then after a couple of times, that particular route seemed like it felt easy, right? It’s night and day difference [from running inside] because you have different types of terrain, weather and wind patterns. You know what I mean?... It gets you”.*

Several participants described engaging in more unstructured, outdoor activities than usual because of the pandemic. Allan highlighted how he initially tried a workout app to help maintain his gym routine, but quickly learnt that it was not a substitute that would work for him. He later explained how the yard work he incorporated was surprisingly “rigorous” and how he lost track of time spent gardening,

*“I found [yard work] was the most significant substitute for my usual gym… I found myself more sore or felt like I got a really good workout in from doing yard work more than any of my gym workouts”.*

The change in routines also provided some participants with opportunities to explore their city and local outdoor surroundings in ways they had not done prior to the pandemic. Madison explained how she started using parks, pathways and visited the mountains surrounding the city,

*“It’s a good reset making you realize that you don’t need to go to the gym to exercise... in Calgary, we can go to the mountains, I can go to Nose Hill Park, or I can bike around”.*

Ana also described how her family began going to the mountains and camping more often. She further explained how she began exploring new and different parks across the city with her family “because [they] can’t go anywhere else”.

Some participants mentioned that they learned more about the benefits of physical activity and also reflected on the enjoyment that resulted from trying new activities. For example, as Ana described,

*“Exercise is important. If we stay on the course and watch screens, [it] makes us grumpy. [My kids and I] learned we need to walk or move our body to stay mentally healthy. Because we didn’t need to try if did everyday routine, my kids were moving. But now we need to play to move our body.”*

Madison also mentioned the enjoyment experienced from her new routines,

*“… now I am finding the time to slow down and take the time to do exercise I enjoy more now”.*

Engaging in new activities allowed participants to reflect on the purpose they attached to their physical activity. Participants no longer considered physical activity as only a means to achieve health benefits, but also a way to enjoy the additional free time participants experienced due to the pandemic.

Although many participants described how they successfully adapted, others were not able to do so. Both John and Mya spoke about missing their aquatic activities and how they were unable to find a suitable substitution for this activity. For example, Mya commented,

*“I use [the] pool for aquacise because I have issues with my knee. I have osteoarthritis, so it’s easier to do those kinds of exercises for cardio... missing the swimming… I really miss that”.*

### 3.3. Theme #3: Balancing Health

Finding the balance between protecting oneself from COVID-19 and complying with pandemic restrictions while maintaining mental health was a significant challenge for many participants. Fears, worries and anxieties towards the pandemic were expressed. Simultaneously, participants offered deeper reflections on how the structure of their lives had changed from the onset of the COVID-19 pandemic and what enabled them to ‘stay healthy’ during this time.

#### 3.3.1. Navigating Different Dimensions of Health during COVID-19

Participants experienced fear related to COVID-19, frequently commenting on the anxieties that they had about the pandemic, becoming infected or contributing to the spread of the virus. Most participants also experienced exhaustion from trying to comply with public health emergency measures. As Benjamin, an older participant, explained,

*“I’m certainly more tired during the day than I was before. I think its part mentally tired as well. When you get tired, you can be short with people… when you’re going to work every day and constantly reminding people of the distancing thing. Constantly washing your hands, and probably 10 times a day more than you would have in the past. Every time you touch something… you wonder if this is it”.*

The interconnections between physical, mental, emotional, and social health have been magnified by the pandemic as participants described challenges related to navigating these different dimensions of health. Christy shared the following,

*“Emotionally or mentally, it’s definitely not without its challenges. I find myself getting mad at the kids more because they’re not being quiet enough for me to work from home... I feel really guilty as a mom… that stress weighs on me a lot… Mental wellbeing I’m not quite where I want to be and it isn’t without its roller coaster. But the physical side I’d say we’re doing really well”.*

Similarly, Jason described how recreation facility closures due to COVID-19 impacted his social well-being,

*“I think the social portion of it… playing badminton… you’re meeting people… Obviously the gyms are opened up to a certain extent, but there’s a lot of people who are scared... I don’t see [my friends] anymore… My overall health hasn’t been [affected], during this COVID timeframe… I do miss some of the social aspects of what I had before”.*

Almost all participants described the struggles of feeling socially isolated and lacking connection with others as negatively impacting their health and well-being.

#### 3.3.2. COVID-19: A Time for Re-Balancing

An unexpected finding was how participants highlighted the pandemic as a positive opportunity to create more balance in their lives. Participants discussed letting go of the pressures that they felt to be productive and questioned the hecticness of their pre-pandemic lifestyles. As Beverly highlighted,

*“It’s made me really rethink, why do we feel like we need to be busy all the time? It’s made me rethink of how much I keep myself occupied even during non-quarantine times. When I go back, am I going to value rest? I have to. It’s going to make me really value rest, and probably make myself less busy”.*

Others expressed concern about going back to their pre-pandemic lifestyles and the challenges that this would place on maintaining a “healthy” sense of balance. As Christy explained,

*“All in all, we actually prefer this… the mornings are low stress… We don’t have to dress up for work anymore. The kids don’t have to get dressed up to go to daycare so there’s a lot of days where they’re left in their pajamas and that’s okay with us. It usually consists a little bit better of family time in the morning, some snuggles, some TV watching… We’re able to really enjoy cooking and preparing meals as a family. Bedtime is less rushed it feels we’re able to breathe a little bit more and enjoy time in our house”.*

Engaging in reflection on how to stay healthy throughout the pandemic was a consistent pattern across interviews. Many participants also noted how their privilege provided protection from the negative impact of the pandemic while others may not be so fortunate. As Allan, who had recently lost his job prior to the COVID-19 pandemic, mentioned,

*“Although I’ve gotten laid off as I’m sure many others have, I’m financially able to do whatever I want… most people aren’t as fortunate… I’ve had [the] ability to be flexible and not be stressed or concerned about things others would be”.*

Christy, who faced numerous challenges while juggling work and parenting at home, also mentioned feeling grateful,

*“I know that we’re maybe the exception versus the rule... a lot of people maybe don’t have the same access to sporting equipment or two parents that are still bringing in an income. We’re very aware that we’re benefiting where most people aren’t and not taking that for granted”.*

### 3.4. Theme #4: Family Life

Participants expressed concerns and challenges related to parenting, and their children’s lack of social connection and increased sedentary behaviour during the pandemic.

#### Challenges for Families

Working from home while parenting was a challenge for parents of young children as Christy, a parent of two pre-school-aged children commented,

*“The biggest challenge is still trying to be a parent while working… not only am I trying to balance a very demanding work where I’m on phone calls or video chats for hours on hours, I have young kids that can’t be left to their own devices all the time… I’m noticing a regression in my children since they’ve been taken out a daycare, so that’s a personal struggle that I’m dealing with”.*

Ana, a mother of two school-aged children, discussed the impacts that the temporary cancellation of youth sports programs had on her son. While she noted this in the context of declines in physical activity, she was primarily concerned about the lack of social interaction he was experiencing. Allan also noted that, “there’s definitely a noticeable mental impact” on both of his daughters at home. John observed the extensive time his post-secondary children were spending on screens,

*“We’ve got to tell them to get out. “Don’t you miss your friends?” No, they see everyone every single day… They look at the computer screen with six different people on there, and everybody’s talking as though they’re in the same room. And they’ll do that for hours on end”*

The experiences of participants with young children during the pandemic appears to be a double-edged sword, with both parenting struggles and appreciation of the increased family time. Amidst the stress of parenting while working full time from home, Christy described the advantages to having her kids at home in relation to her work–life balance,

*“It is difficult with the kids at home, but I wouldn’t do the latter [go back to her pre-COVID-19 lifestyle]. The work-life balance was really hard. Now that we’ve settled into a newer normal, we’ve got our routines figured out”.*

## 4. Discussion

The COVID-19 pandemic has impacted daily routines related to work, school, home and family life, socializing, and leisure activity. Our findings demonstrate both positive and negative effects of the pandemic on physical activity and perceptions of health. Notably, many participants developed strategies to maintain their pre-pandemic physical activity levels. These adaptations were unique to each individual’s circumstances and motivation, such as their beliefs about the benefits of a physically active lifestyle. Adapting routines also facilitated individual reflection, with many participants reporting insights about the unexpected positive impacts that occurred due to the pandemic.

In support of previous studies in Canada [21] and elsewhere [22,23,24,25,49,50], we found that participants have experienced changes in their physical activity due to the pandemic. Our novel findings highlight that physical activities which could no longer be undertaken due to the public health restrictions were replaced by other types of physical activity. For example, participants replaced activities that they would normally undertake at fitness and recreation facilities, with alternatives, often involving activity outdoors (e.g., walking or cycling) and at home. Moreover, the pandemic encouraged many participants to try new physical activities that they may never have attempted (e.g., skateboarding or hiking). Although some participants modified their physical activity routines during the pandemic, others had difficulty adapting. Behaviour change models, such as the Theory of Planned Behaviour [31], may help explain people’s ability to adapt their physical activity routines during the pandemic. For example, participants who successfully adapted their routines may have had higher self-efficacy related to physical activity [31,51] and therefore were able to overcome barriers they faced during the pandemic. In support, Lesser and Nienhuis [52] found reductions in physical activity to be greater among those who were inactive prior to the pandemic relative to those who were already active.

For some participants, finding suitable substitutes for physical activities during the pandemic was difficult when their preferred activity choices required access to specific infrastructure like swimming pools and exercise facilities. Others found it difficult to replace their usual gym routines with exercise apps or at-home fitness equipment and thus, never found an appropriate alternative activity during the pandemic. Non-pandemic studies have found availability and access to local recreational facilities is important for supporting leisure physical activity [53,54]. For example, the sudden temporary closure of a university recreational facility resulted in challenges for users trying to find alternative physical activities that otherwise required activity-specific infrastructure [55]. Access to local recreational facilities can reduce motivational barriers to physical activity [56]. During public health restrictions that involve closures of recreational facilities, concurrent strategies that provide alternative local outdoor opportunities (e.g., skating rinks, road closures supporting pedestrians and cyclists) might be needed to help adults maintain regular physical activity.

Although fitness and recreation facilities were re-opened during our study, many participants had not returned to using them. A contributing explanation may be that the re-opening of fitness and recreation facilities occurred during summer, and many participants found outdoor alternatives to their usual indoor physical activities (e.g., parks and using pathways for walking and biking). Some participants commented they would return to using indoor facilities only if appropriate physical distancing measures were implemented, while others mentioned they may not return to these facilities as they appreciated their new exercise routines. There is some, albeit limited, evidence suggesting that some users of sports and recreational clubs which closed due to the pandemic may not return to these clubs after their re-opening [57]. Furthermore, the pandemic might impact the types and intensity of physical activity people undertake during the pandemic because of different exercise locations [58]. Our findings highlight the importance of public outdoor places and spaces for supporting physical activity during pandemics, including the need for these locations to remain accessible when private recreational facilities are forced to close under public health restrictions. The increased use of outdoor public recreational facilities during the pandemic may also require additional measures to ensure physical distancing is maintained. Indoor, home-based alternatives (e.g., online and home fitness activities) provide another option for maintaining physical activity when fitness and recreation facilities are forced to close [27].

A perceived lack of time is a consistently reported barrier to participating in leisure-time physical activity [56,59]. Several studies suggest that sociodemographic characteristics (e.g., age, family type, relationship status, children, and employment status) are associated with a shortage of physical activity time [56,59]. In our study, participants reported that the pressures of scheduling physical activity into daily routines were alleviated by having more time as an unexpected benefit due to the pandemic. Related to this extra time, participants were able to undertake more outdoor, unstructured, forms of physical activity for the enjoyment and escape it provided, and not exclusively to obtain health benefits. Leisure-time activities are predominantly considered an intrinsically enjoyable experience that can result in improved mental well-being [60], increased self-fulfillment [61], and reduced negative psychological states [62]. Similar to other studies [63], we found the COVID-19 pandemic had a negative impact on the mental health of some individuals. Kaur et al. [6] found that alternative exercise arrangements adults made due to COVID-19, not only contributed to maintaining their exercise routines, but these modifications also contributed to the maintenance of their physical and mental health. Coping strategies are desperately needed to counteract the negative consequences of the COVID-19 public health restrictions on mental health [63]. Encouraging individuals to find physical activities that provide intrinsic enjoyment could improve mental health [60,62,64] and support successful adaptation to new physical activity routines during the pandemic.

Participants discussed how their lives were easier during the pandemic because they had the financial means to purchase exercise equipment or be active outdoors (e.g., purchase rain gear to be active outdoors in all conditions). Financial pressures due to COVID-19 caused anxiety in other individuals [65]. Our findings highlight that higher socioeconomic status may protect some individuals in terms of their physical activity and health levels. This may be one pathway through which the pandemic exacerbates social and health inequalities. Studies conducted in the United States [66] and Sweden [67] have reported higher rates of COVID-19 cases and mortality amongst groups with a lower socioeconomic status (i.e., lower education level) and racial minorities. Governments and health authorities need to consider how public health restrictions are contributing to unequal social, health, financial and economic experiences during the pandemic [68].

To support the development of effective public health policies and programs during the COVID-19 pandemic, research strategies that recognize the deeply complex and contextual nature of health issues are necessary [69]. The constructivist paradigm underpinning this study is based on the belief that reality is socially constructed and thus, emphasizes the interconnections between social-cultural context and lived experiences [70]. Use of this paradigm for our study has supported a more holistic understanding of the ways in which COVID-19 and the related public health restrictions are affecting different dimensions of health. Our study findings provide a starting point to informed health promotion strategies during pandemics. Further, the results can help to engage communities in developing contextually relevant solutions to the health issues that they are facing as a result of the COVID-19 pandemic public health restrictions. As the public health community continues to respond and investigate the various influences of the COVID-19 pandemic on health, physical activity, and sedentary behaviour, it is important to recognize that alternative paradigms, including constructivist approaches, can offer a valuable approach to understanding complex issues [71].

### Limitations

Our study has several limitations. Interviews were conducted in summer, and despite asking participants to reflect on their experiences since the pandemic declaration (in late winter), seasonality might have influenced responses related to physical activity. Our study captures experiences within the first four months of the pandemic and thus may not represent the current experiences related to the ongoing pandemic. Future research should consider seasonality, especially in climates that have harsh winters that may be a barrier to outdoor activity during COVID-19. Our sampling frame was based on participants who previously completed an online community survey in Calgary [40], which may influence the range of experiences and perceptions reported. For example, our findings might not reflect the experiences of those most socioeconomically disadvantaged. Moreover, those who respond to health surveys tend to be healthier and more motivated compared to non-respondents [72]. Finally, contextual differences in the timing and degrees to which public health measures were implemented [73,74], access and availability of public infrastructure to support physical activity [75,76] and socioeconomic conditions [77,78] would likely limit the transferability [40] of our results to other settings.

## 5. Conclusions

Our findings suggest that encouraging outdoor physical activity in public spaces and places (with physical distancing restrictions) is important for supporting active lifestyles and promoting physical and mental health during the pandemic. Targeted and contextually relevant strategies may also be needed to encourage physical activity and reduce sedentary behaviour. Understanding ways to support positive adaptation processes during the changing public health restrictions is vital for developing interventions to enhance health and physical activity during the pandemic. Given the health benefits of physical activity [14], further research on the effectiveness of intervention strategies that support physical activity during the COVID-19 pandemic should be prioritized.

## Data Availability

The data are not publicly available due to ethical approvals at the time.

## References

[1] World Health Organization Director–General’s Opening Remarks at the Media Briefing on COVID-19. https://www.who.int/director-general/speeches/detail/who-director-general-s-opening-remarks-at-the-media-briefing-on-covid-19---11-march-2020.

[2] World Health Organization Coronavirus (COVID-19) Dashboard: Situation by Country, Territory and Area. https://covid19.who.int/table.

[3] Haug N., Geyrhofer L., Londei A., Dervic E., Desvars-Larrive A., Loreto V., Pinior B., Thurner S., Klimek P. (2020). Ranking the effectiveness of worldwide COVID-19 government interventions. Nat. Hum. Behav..

[4] Girum T., Lentiro K., Geremew M., Migora B., Shewamare S. (2020). Global strategies and effectiveness for COVID-19 prevention through contact tracing, screening, quarantine, and isolation: A systematic review. Trop. Med. Health.

[5] Alberta Government CMOH Order 02-2020: 2020 COVID-19 Response. https://open.alberta.ca/publications/cmoh-order-02-2020-2020-covid-19-response.

[6] Kaur H., Singh T., Arya Y.K., Mittal S. (2020). Physical Fitness during the Covid-19 Pandemic: A Qualitative Enquiry. Front. Psychol..

[7] Sallis R., Young D.R., Tartof S.Y., Sallis J.F., Sall J., Li Q., Smith G.N., Cohen D.A. (2021). Physical inactivity is associated with a higher risk for severe COVID-19 outcomes: A study in 48,440 adult patients. Br. J. Sports Med..

[8] Chen P., Mao L., Nassis G.P., Harmer P., Ainsworth B.E., Li F. (2020). Coronavirus disease (COVID-19): The need to maintain regular physical activity while taking precautions. J. Sport Health Sci..

[9] Stockwell S., Trott M., Tully M., Shin J., Barnett Y., Butler L., McDermott D., Schuch F., Smith L. (2021). Changes in physical activity and sedentary behaviours from before to during the COVID-19 pandemic lockdown: A systematic review. BMJ Open Sport Exerc. Med..

[10] Cunningham C., O’Sullivan R., Caserotti P., Tully M.A. (2020). Consequences of physical activity in older adults: A systematic review of reviews and meta-analyses. Scand. J. Med. Sports.

[11] Lippi G., Henry B.M., Sanchis-Gomar F. (2020). Physical inactivity and cardiovascular disease at the time of coronavirus disease 2019 (COVID-19). Eur. J. Prev. Cardiol..

[12] Mujika I., Padilla S. (2000). Detraining: Loss of training-induced physiological and performance adaptations. Part I: Short term insufficient training stimulus. Sports Med..

[13] Mujika I., Padilla S. (2001). Cardiorespiratory and metabolic characteristics of detraining in humans. Med. Sci. Sports Exerc..

[14] Anderson E., Shivakumar G. (2013). Effects of exercise and physical activity on anxiety. Front Psych..

[15] Callaghan P. (2004). Exercise: A neglected intervention in mental health care. J. Psychiatr. Ment. Health Nurs..

[16] Warburton D., Nicol C., Bredin S. (2006). Health benefits of physical activity: The evidence. Can. Med. Assoc. J..

[17] Nieman D.C., Wentz L.M. (2019). The compelling link between physical activity and the body’s defense system. J. Sport Health Sci..

[18] Grande A.J., Keogh J., Silva V., Scott A.M. (2020). Exercise versus no exercise for the occurrence, severity, and duration of acute respiratory infections. Cochrane Database Syst. Rev..

[19] Nieman D.C. (2020). Coronavirus disease-2019: A tocsin to our aging, unfit, corpulent, and immunodeficient society. J. Sport Health Sci..

[20] Bull F.C., Al-Ansari S.S., Biddle S., Borodulin K., Buman M.P., Cardon G., Carty C., Chaput J.P., Chastin S., Chou R. (2020). World Health Organization 2020 guidelines on physical activity and sedentary behaviour. Br. J. Sports Med..

[21] Rhodes R.E., Liu S., Lithopoulos A., Garcia-Barrera M.A. (2020). Correlates of perceived physical activity transitions during the COVID-19 pandemic among Canadian adults. Appl. Psychol. Health Well-Being.

[22] Cheval B., Sivaramakrishnan H., Maltagliati S., Fessler F., Forestier C., Sarrazin P., Orsholits D., Chalabaev A., Sander D., Ntoumanis N. (2020). Relationships between changes in self-reported physical activity, sedentary behaviour and health during the coronavirus (COVID-19) pandemic in France and Switzerland. J. Sports Sci..

[23] Qin F., Song Y., Nassis G.P., Zhao L., Dong Y., Zhao C., Feng Y., Zhao J. (2020). Physical activity, screen time, and emotional well-being during the 2019 novel coronavirus outbreak in China. Int. J. Environ. Res. Public Health.

[24] Bhutani S., Cooper J.A., Vandellen M.R. (2020). Self-reported changes in energy balance behaviors during COVID-19 related home confinement: A cross-sectional study. medRxiv.

[25] Chtourou H., Trabelsi K., H’mida C., Boukhris O., Glenn J.M., Brach M., Bentlage E., Bott N., Shephard R.J., Ammar A. (2020). Staying physically active during the quarantine and self-isolation period for controlling and mitigating the COVID-19 pandemic: A systematic overview of the literature. Front. Psychol..

[26] Gropper H., John J.M., Sudeck G., Thiel A. (2020). The impact of life events and transitions on physical activity: A scoping review. PLoS ONE.

[27] Goethals L., Barth N., Guyot J., Hupin D., Celarier T., Bongue B. (2020). Impact of home quarantine on physical activity among older adults living at home during the COVID-19 pandemic: A qualitative interview study. J. Med. Internet. Res. Aging.

[28] Hilliard M.E., Riekert K.A., Ockene J.K., Pbert L. (2018). The Handbook of Health Behaviour.

[29] Rosenstock I.M. (1974). Historical origins of the health belief model. Health Educ. Monogr..

[30] Prochaska J.O., DiClemente C.C., Norcross J.C., Goldfield M.R. (2005). The transtheoretical approach. Handbook of Psychotherapy Integration.

[31] Ajzen I. (1991). The theory of planned behaviour. Organ. Behav. Hum. Decis. Process..

[32] Janevic M.R., Connell C.M., Hilliard M.E., Riekert K.A., Ockene J.K., Pbert L. (2018). Individual theories. The Handbook of Health Behaviour.

[33] Sallis J., Bauman A., Pratt M. (1998). Environmental and policy interventions to promote physical activity. Am. J. Prev. Med..

[34] Spence J.C., Lee R.E. (2003). Toward a comprehensive model of physical activity. Sport Exerc..

[35] Fitzgibbon M.L., Buscemi J., Cory M., Jagpal A., Brush B., Kong A., Tussing-Humphreys L., Hilliard M.E., Riekert K.A., Ockene J.K., Pbert L. (2018). Understanding population health from multilevel and community-based models. The Handbook of Health Behaviour.

[36] Myer R.A., Moore H.B. (2011). Crisis in context theory: An ecological model. J. Couns. Dev..

[37] Holt N., Smith B., Sparkes A.C. (2016). Doing grounded theory in sport and exercise. Routledge Handbook of Qualitative Research in Sport and Exercise.

[38] Charmaz K. (2004). Reconstructing theory in grounded theory studies. Constructing Grounded Theory: A Practical Guide through Qualitative Analysis.

[39] Sbaraini A., Carter S.M., Evans R.W., Blinkhorn A. (2011). How to do a grounded theory study: A worked example of a study of dental practices. BMC Med. Res. Methodol..

[40] McCormack G.R., Doyle-Baker P.K., Petersen J.A., Ghoneim D. (2020). Parent anxiety and perceptions of their child’s physical activity and sedentary behavior during the COVID-19 pandemic in Canada. Prev. Med. Rep..

[41] Glesne C. (2016). Becoming Qualitative Researchers: An Introduction.

[42] Palinkas L.A., Horwitz S.M., Green C.A., Wisdom J.P., Duan N., Hoagwood K. (2015). Purposeful sampling for qualitative data collection and analysis in mixed method implementation research. Adm. Policy Ment. Health.

[43] Jones S.R., Torres V., Arminio J. (2014). Negotiating the Complexities of Qualitative Research in Higher Education: Fundamental Elements and Issues.

[44] Hall W., Callery P. (2001). Enhancing the rigour of grounded theory: Incorporating reflexivity and relationality. Qual. Health Res..

[45] Opening Soon—Alberta’s Relaunch Strategy. https://open.alberta.ca/dataset/61f54c09-d6d7-4a12-a5be-0bc663a02c31/resource/e158ff14-eab7-4f24-94f4-b67c3639d0d5/download/covid-19-alberta-relaunch-strategy-2020-06.pdf.

[46] Braun V., Clark V. (2019). To saturate or not to saturate? Questioning data saturation as a useful concept for thematic analysis and sample size rationales. Qual. Res. Sport Exerc. Health.

[47] Braun V., Clarke V. (2006). Using thematic analysis in psychology. Qual. Res. Psychol..

[48] (2018). QSR International Pty Ltd. NVivo (Version 12). https://www.qsrinternational.com/nvivo-qualitative-data-analysis-software/home.

[49] Flanagan E.W., Beyl R.A., Fearnbach S.N., Altazan A.D., Martin C.K., Redman L.M. (2020). The impact of COVID-19 stay-at-home orders on health behaviours in adults. Obesity.

[50] Stanton R., To Q.G., Khalesi S., Williams S.L., Alley S.J., Thwaite T.L. (2020). Depression, anxiety and stress during COVID-19: Associations with changes in physical activity, sleep, tobacco and alcohol use in Australian adults. Int. J. Environ. Res. Public Health.

[51] Bandura A. (1997). Self-Efficacy: The Exercise of Control.

[52] Lesser I.A., Nienhuis C.P. (2020). The impact of COVID-19 on physical activity behaviour and well-being of Canadians. Int. J. Environ. Res. Public Health.

[53] Gidlow C., Cerin E., Sugiyama T., Adams M.A., Mitas J., Akram M., Reis R.S., Davey R., Troelsen J., Schofield G. (2019). Objectively measured access to recreational destinations and leisure-time physical activity: Associations and demographic moderators in a six-country study. Health Place.

[54] Farneti C., Ditch D. (2018). The impact of an unexpected facility closure on a campus community. Recreat. Sports J..

[55] Cerin E., Leslie E., Sugiyama T., Owen N. (2010). Perceived barriers to leisure-time physical activity in adults: An ecological perspective. J. Phys. Act. Health.

[56] Sowier-Kasprzyk I., Widawska-Stanisz A. (2020). Changes in attitudes of consumers of sports and recreational services in the context of COVID-19. J. Phys Educ. Sport..

[57] Dunton G.F., Wang S.D., Do B., Courtney J. (2020). Early effects of the COVID-19 pandemic on physical activity locations and behaviors in adults living in the United States. Prev. Med. Rep..

[58] Borodulin K., Sipilä N., Rahkonen O., Leino-Arjas P., Kestila L., Jousilahti P., Prattala R. (2016). Socio-demographic and behavioral variation in barriers to leisure-time physical activity. Scand. J. Public Health.

[59] Cheng E., Pegg S. (2016). “If I’m not gardening, I’m not at my happiest”: Exploring the positive subjective experiences derived from serious leisure gardening by older adults. World Leis. J..

[60] Stebbins R.A. (2006). Leisure studies: The happy science. Leis. Stud. Assoc. Newsl..

[61] Dupuis S.L., Smale B.J.A. (2013). An examination of relationship between psychological well-being and depression and leisure activity participation among older adults. Soc. Leis..

[62] Ammar A., Trabelsi K., Brach M., Chtourou H., Boukhris O., Masmoudi L., Bouaziz B., Bentlage E., How D., Ahmed M. (2020). Effects of home confinement on mental health and lifestyle behaviours during the COVID-19 outbreak: Insight from the ECLB-COVID19 multicenter study. Biol. Sport.

[63] Javed B., Sarwer A., Soto E.B., Mashwani Z.R. (2020). The coronavirus (COVID-19) pandemic’s impact on mental health. Int. J. Health Plan. Manag..

[64] Fuzeki E., Groneberg D.A., Banzer W. (2020). Physical activity during COVID-19 induced lockdown: Recommendations. J. Occup. Med. Toxicol..

[65] Mann F.D., Krueger R.F., Vohs K.D. (2020). Personal economic anxiety in response to COVID-19. Pers. Individ. Differ..

[66] Hawkins R.B., Charles E.J., Mehaffey J.H. (2020). Socio-economic status and COVID-19-related cases and fatalities. Public Health.

[67] Strange P., Furst P., Schultz T. (2020). Excess deaths from COVID-19 correlate with age and socio-economic status: A database study in the Stockholm region. Upsala J. Med. Sci..

[68] Bambra C., Riordan R., Ford J., Matthews F. (2020). The covid-19 pandemic and health inequalities. J. Epidemiol. Community Health.

[69] Tremblay M.C., Richard L. (2011). Complexity: A potential paradigm for health promotion discipline. Health Promot. Int..

[70] Labonte R., Robertson A. (1996). Delivering the goods, showing our stuff: The case for a constructivist paradigm for health promotion research and practice. Health Educ. Behav..

[71] Guba E.G., Lincoln Y.S., Denzin N.K., Lincoln Y.S. (2005). Paradigmatic controversies, contradictions, and emerging confluences. Handbook of Qualitative Research.

[72] Holle R., Hochadel M., Reitmeir P., Meisinger C., Wichman H.E. (2006). Prolonged recruitment efforts in health surveys: Effects on response, costs, and potential bias. Epidemiology.

[73] Islam N., Sharp S.J., Chowell G., Shabnam S., Kawachi I., Lacey B., Massaro J.M., D’Agostino Sr R.B., White M. (2020). Physical distancing interventions and incidence of coronavirus disease 2019: Natural experiments in 149 countries. BMJ.

[74] Vinceti M., Filippini T., Rothman K.J., Ferrari F., Goffi A., Maffeis G., Orsini N. (2020). Lockdown timing and efficacy in controlling COVID-19 using mobile phone tracking. EClinicalMedicine.

[75] Owen N., Humpel N., Leslie E., Bauman A., Sallis J.F. (2004). Understanding environmental influences on walking: Review and research agenda. Am. J. Prev. Med..

[76] Panter J., Heinen E., Mackett R., Ogilvie D. (2016). Impact of new transport infrastructure on walking, cycling, and physical activity. Am. J. Prev. Med..

[77] Ball K., Salmon J., Giles-Corti B., Crawford D. (2006). How can socio-economic differences in physical activity amongst women be explained? A qualitative study. Women Health.

[78] Cerin E., Leslie E. (2008). How socio-economic status contributes to participation in leisure-time physical activity. Soc. Sci. Med..

